# Supplementary material for: Exploring the most promising anti ‐ Depressant drug targeting Microtubule Affinity Receptor Kinase 4 involved in Alzheimer’s Disease through molecular docking and molecular dynamics simulation
Source: PLoS One. 2024 Jul 25;19(7):e0301179. doi: 10.1371/journal.pone.0301179 (PMC11271900; doi:10.1371/journal.pone.0301179)
Supplement: S1 Fig — H-bond occupancy plot of the protein 5SE1 in complex with (A) 4184, (B) 2771, (C) 4205, (D) 5533, (E) 4543 (F) 2160 (G) 3152. (DOCX) [file pone.0301179.s001.docx]

**Exploring The Most Promising Anti - Depressant Drug Targeting Microtubule Affinity Receptor Kinase 4** I**nvolved In Alzheimer’s Disease Through Molecular Docking and Molecular Dynamics Simulation**

**SUPPLEMENTARY 1**


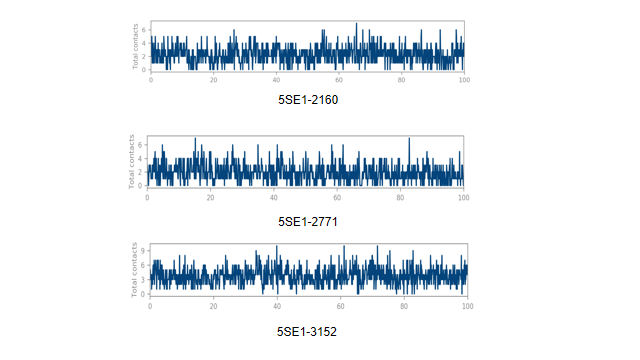


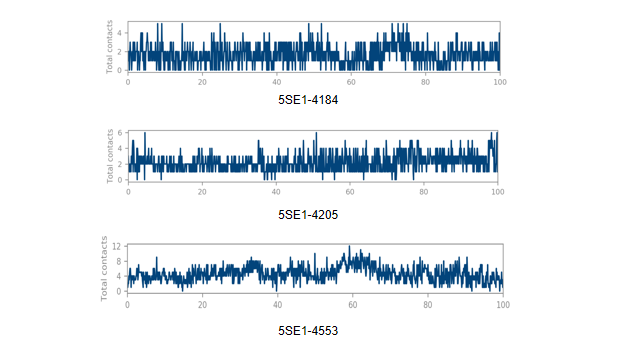


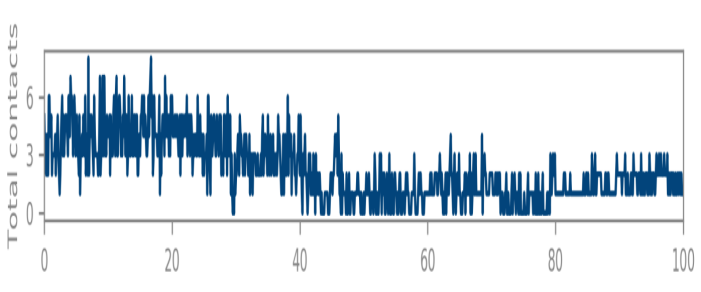


**Supplementary Figure 1-** H-bond occupancy plot of the protein 5SE1 in complex with (A) 4184, (B) 2771, (C) 4205, (D) 5533, (E) 4543 (F) 2160 (G) 3152
