# Supplementary material for: Exploring the most promising anti ‐ Depressant drug targeting Microtubule Affinity Receptor Kinase 4 involved in Alzheimer’s Disease through molecular docking and molecular dynamics simulation
Source: PLoS One. 2024 Jul 25;19(7):e0301179. doi: 10.1371/journal.pone.0301179 (PMC11271900; doi:10.1371/journal.pone.0301179)
Supplement: S1 Table — (DOCX) [file pone.0301179.s002.docx]

| **Drug Name** | **Molecular weight g/mol** | **Pharmacokinetics** | | | **Druglikeness** |
| --- | --- | --- | --- | --- | --- |
|  |  | **GI absorption** | **BBB permeant** | **Log *K*_p_   cm/s** | **Lipinski** |
| Sertraline | 306.23 | High | Yes | -4.77 | Yes; 1 violation: MLOGP>4.15 |
| Fluoxetine | 309.33 | High | Yes | -5.18 | Yes; 0 violation |
| Escitalopram | 324.39 | High | Yes | -5.99 | Yes; 0 violation |
| Fluvoxamine | 318.33 | High | Yes | -6.37 | Yes; 0 violation |
| Paroxetine | 329.37 | High | Yes | -5.82 | Yes; 0 violation |
| Citalopram | 324.39 | High | Yes | -5.99 | Yes; 0 violation |
| Agomelatine | 243.3 | High | Yes | -5.85 | Yes; 0 violation |
| Mianserine | 264.36 | High | Yes | -5.51 | Yes; 0 violation |
| Reboxetine | 313.39 | High | Yes | -6.07 | Yes; 0 violation |
| Trazodone | 371.86 | High | Yes | -6.55 | Yes; 0 violation |
| Venlafaxine | 277.4 | High | Yes | -8.65 | Yes; 0 violation |
| Bupropion | 239.74 | High | Yes | -5.48 | Yes; 0 violation |
| Duloxtine | 297.41 | High | Yes | -5.05 | Yes; 0 violation |
| Mirtazapine | 265.35 | High | Yes | -6.04 | Yes; 0 violation |
| Tranylcypromine | 133.19 | High | Yes | -6.05 | Yes; 0 violation |
| Moclobemide | 268.74 | High | Yes | -6.88 | Yes; 0 violation |
| Phenelzine | 136.19 | High | Yes | -6.31 | Yes; 0 violation |
| Isocarboxazid | 231.25 | High | Yes | -6.65 | Yes; 0 violation |
| Amitriptyline | 277.4 | High | Yes | -4.41 | Yes; 1 violation: |
| Trimipramine | 294.43 | High | Yes | -3.98 | Yes; 0 violation |
| Imipramine | 280.41 | High | Yes | -4.6 | Yes; 0 violation |
| Clomipramine | 314.85 | High | Yes | -4.54 | Yes; 1 violation: |
| Dosulepin | 295.44 | High | Yes | -4.91 | Yes; 1 violation: |
| Nortriptyline | 263.38 | High | Yes | -4.7 | Yes; 0 violation |

**SUPPLEMENTARY 2**
