# Supplementary material for: Exploring the most promising anti ‐ Depressant drug targeting Microtubule Affinity Receptor Kinase 4 involved in Alzheimer’s Disease through molecular docking and molecular dynamics simulation
Source: PLoS One. 2024 Jul 25;19(7):e0301179. doi: 10.1371/journal.pone.0301179 (PMC11271900; doi:10.1371/journal.pone.0301179)
Supplement: S1 Dataset — (DOCX) [file pone.0301179.s003.docx]

Minimal Data Set Definition

Based on the provided methodology and results, the minimal data set required to replicate the study findings reported in your manuscript would include the following:

1. **Data from ADMET Analysis:**

Results of the ADMET analysis for the 24 SSRI drugs performed using the SWISS ADME server, including parameters such as pharmacokinetics and drug likeliness profile.

1. **Molecular Docking Data:**

Docking results for all 24 SSRI drugs with MARK4 protein, including the binding energies and interactions observed.

Specifically, for the top six drugs (CID ID - 4184, 2771, 4205, 5533, 4543, and 2160), provide detailed information on the binding modes, including any hydrogen bond interactions, salt bridges, and other relevant interactions with the active site residues of MARK4.

1. **Molecular Dynamics Simulation Data:**

Data from molecular dynamics simulations conducted for the six selected SSRI drugs and donepezil (CID ID-3152), including simulation trajectories and analysis results.

Information regarding the conformational changes, stability, and interactions observed during the simulations, particularly focusing on the behaviour of the ligands in complex with MARK4 over the simulation period.

Any post-trajectory analysis results such as free energy landscape (FEL), polarizable continuum model (PCM), and MMGBSA calculations.

1. **MMGBSA Calculations Data:**

Results of the Molecular Mechanics Generalised Born Surface Area (MMGBSA) calculations for the anchored complexes, including the binding free energies (∆Gobind) calculated for each ligand-protein complex.

1. **Supplementary Materials:**

Supplementary materials containing relevant figures, plots, and additional details supporting the findings presented in the manuscript, such as radar plots illustrating the pharmacokinetics and binding affinities of the top six drugs.
